# Experimental Evolution of Pathogenesis: “Patient” Research

**DOI:** 10.1371/journal.ppat.1003340

**Published:** 2013-05-30

**Authors:** Alexander W. Ensminger

**Affiliations:** 1 Department of Molecular Genetics, University of Toronto, Toronto, Ontario, Canada; 2 Public Health Ontario, Toronto, Ontario, Canada; Duke University Medical Center, United States of America

## Overview

**Figure 1:**
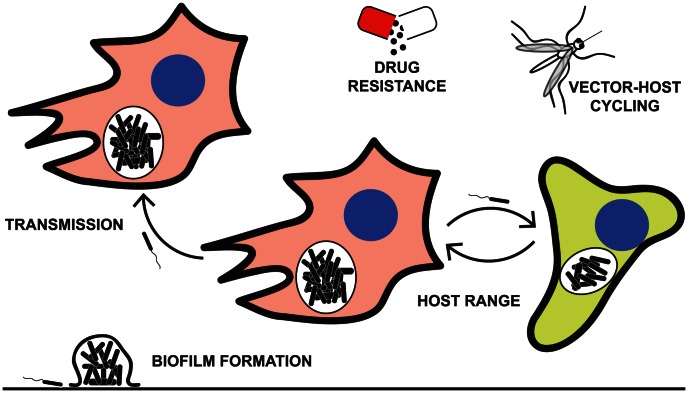
Some of the pathogenic characteristics explored by laboratory passage models. Examples of the application of experimental evolution to the study of pathogens include modeling the evolution of drug resistance [18]–[20], trade-offs between generalist versus specialist adaptations to the host [9], [12], pathogen transmission [13], [14], and biofilm formation [17].

Twenty-five years and over 50,000 bacterial generations ago, one of the longest open-ended experiments in modern times began when the Lenski laboratory started passaging *Escherichia coli* under conditions of limited glucose as a carbon source [1]. Later that same year, a paper was published describing a new method for DNA sequencing, called pyrosequencing, that would ultimately light a path toward massively parallel (next-generation) sequencing [2]. As of 2013, a raw megabase of DNA costs less than $0.10 [3], or roughly equivalent to the cost of one petri dish. Combined with access to this affordable, high-throughput sequencing, experimental evolution [4] represents a sophisticated approach to dissecting host-pathogen interactions and testing models of host range and pathogen adaptation. Here, we review how experimental evolution has been used to directly test models of host-pathogen interactions (Figure 1). We discuss parallels between these laboratory studies and the real-world evolutionary trajectories that have been uncovered through the direct sequencing of clinical isolates.

## Experimental Adaptation of Host Range

Laboratory passage has long been recognized as an effective means to modify the host range of pathogens, with successive passage of viruses in nonhuman hosts an early strategy for generating live attenuated vaccines [5]. The principle behind this attenuation is that confined passage in one host species can modify the host range of a pathogen such that it no longer efficiently causes disease in the original host [6]. Armed with modern molecular and genomic tools, several groups have revisited the basic outlines of this approach to directly test how natural host diversity and host cycling influence the evolutionary trajectory of pathogens.

Why do some pathogens specialize to one particular host while others remain generalists, able to infect a broad range of host species? One model is that exposure to diverse environmental hosts drives the expansion of host range and prevents specialization to any single host [7]. In this model, generalist pathogens fail to specialize because acquired host-specific adaptations likely result in antagonistic pleiotropy [8] in other hosts encountered during the pathogen life cycle, leading to their rapid exclusion from future generations. We recently used this approach to uncover fitness trade-offs in the broad host-range bacterial pathogen *Legionella pneumophila* during its adaptation to mouse macrophages [9]. *L. pneumophila* normally replicates in freshwater amoebae [10], and our model is that its broad host range is maintained due to environmental diversity of amoebae within its natural reservoirs. Consistent with this model, several identified mutations selected for in the macrophage-adapted strains came at the expense of reduced fitness in two natural amoebal hosts [9].

Intuitively, such host dynamics should also shape the genomes of vector-borne diseases, where pathogens must balance fitness in both their vector and their other hosts. This hypothesis has been extensively tested in arthropod-borne viruses, where host cycling between insect vector and mammalian host should prevent the accumulation of mutations that result in fitness costs in either host, perhaps holding such pathogens in a sort of evolutionary stasis [11]. A recent in vivo experimental evolution study, however, showed that the evolution of West Nile virus, when passaged in either chicks or mosquitos, does not strictly fit the trade-off hypothesis [12]. Instead of each host selecting for unique host-specific adaptations, it was suggested that the mosquito host overall placed less stringent selection on the virus, allowing for expanded viral genetic diversity within the vector host. Once these viruses were transferred to the chick host, however, strong positive selection took over, selecting for virions carrying mutations that increased fitness within the vertebrate hosts. It will be particularly interesting to see whether similar complexities exist in the host-pathogen interactions of non–vector-borne diseases, such as *L. pneumophila*. Whether due to less stringent selection under some conditions, disruptions in environmental host diversity, increased mutation, or population bottlenecking, transient changes in pathogen diversity might subsequently influence the outcome of human exposure to these bacteria.

## Experimental Modeling of Other Pathogenic Traits

Experimental evolution can also be used to modulate a pathogen's transmission between hosts. Usually, the conditions of serial passage (back dilution in growth media) in the laboratory do not recapitulate the nuances involved in natural transmission between hosts, selecting instead for mutations that increase within-host replication [6]. Nevertheless, recent work on H5N1 influenza, a deadly but poorly transmitted strain, indicates that this need not always be the case [13], [14]. Two independent groups used experimental evolution to select for transmissibility by serially passaging the virus between ferrets. One early model of host-pathogen interactions would have predicted a trade-off between within-host virulence and between-host transmissibility, but in recent years the universal applicability of this model has been questioned [15]. Indeed, these experimentally adapted viruses retained their ability to efficiently kill ferrets while showing dramatic improvements in their ability to spread through a population of hosts, which is inconsistent with the simple virulence/transmission trade-off model. Due to the recognized overlap between ferret-to-ferret and person-to-person spread, both the publication of these findings and the continuation of this research remain the subject of considerable debate.

In addition to transmission and host-range trade-offs, experimental evolution has also provided unique insight into other aspects of pathogenesis, including the fitness trade-offs between colonization and systemic infection [16], the community architecture during biofilm formation [17], and the development of drug resistance in both prokaryotic and eukaryotic pathogens [18]–[20]. Particular focus has been placed on the predominant bacterial pathogens affecting cystic fibrosis patients, as evolutionary trajectories within each patient are thought to have profound impacts on the severity of disease [21], [22]. Experimental passage of *Pseudomonas aeruginosa* in media designed to mimic the conditions encountered by bacteria in the lungs of cystic fibrosis patients selected for several mutations, including antibiotic resistance, that overlapped with known mutations previously identified in patient isolates [20]. Another study used experimental evolution to model the complex dynamics of biofilm production in a population of *Burkholderia cenocepacia*, another frequent pathogen of cystic fibrosis patients [17]. Rather than selecting for clones that followed one evolutionary trajectory to maximum fitness, careful examination of the population during this passage indicated that multiple types of clones evolved to occupy different environmental niches within the experiment. Several parallels were observed between the adaptations selected for in this in vitro model of biofilm formation and what has been learned from studying clinical isolates from chronic infections of the cystic fibrosis lung, including an increased ability to utilize limited iron and alterations in lipopolysaccharide production.

## Patience versus Patients—Genomic Epidemiology

Many of the concepts of experimental evolution are also relevant to the expanding field of genomic epidemiology, where using whole-genome sequencing to track the spread of disease through a population can also provide real-world information about how pathogens adapt to their human host. In particular, concepts such as parallel genome evolution (repeated selection for mutations in the same genes or pathways in independent clones) and fitness trade-offs between human infection and environmental persistence are as important for understanding outbreaks of disease as they are to laboratory adaptation.

The life-long colonization of cystic fibrosis patients with *Burkholderia dolosa* [21] and *Pseudomonas aeruginosa* [22] has provided a unique window into the evolutionary trajectory of these pathogens during adaptation to the human host. In longitudinal studies of both pathogens, independent lineages acquired mutations in an overlapping set of genes, including *gyrA* (drug resistance) and global response regulators. In *P. aeruginosa* replicating for 200,000 generations, the observation of purifying negative selection suggested that these populations reached fitness peaks not previously observed during in vitro adaptation of the pathogen [22]. In another study that blurred the lines between experimental evolution and genomic epidemiology in patients, the adaptation of an asymptomatic bacteriuria strain of *E. coli* to the human host was monitored by sequencing a series of isolates taken from the bladders of six patients that were deliberately colonized with this strain [23].

## Sequence First and Ask Questions Later

Like many fields, experimental evolution has not escaped the transformative effects of next-generation sequencing. By sequencing the genomes of laboratory-adapted strains, it is now possible to define the molecular basis of microbial adaptation to new environments or conditions. Given that, using current technologies—even large bacterial genomes currently cost less than $100 to sequence at >100× coverage—we favor what could best be described as a *sequence first and ask questions later* approach to understanding the laboratory evolution of pathogens. Indeed, complex population dynamics, including frequent clonal interference between competing clones within a population, are likely to be quite common during these experiments [9], [17]. To capture these dynamics, either several isolates from intermediate time-points should be sequenced, high-resolution genotyping should be performed [9], [17], or metagenomic sequencing of the population should be used to estimate allelic frequencies across the experimental time frame [17], [24].

## What's Next?

With the increased ability to link laboratory adaptations to specific genetic lesions via affordable, widely accessible sequencing, the application of experimental evolution to study pathogen adaptation has never held more potential (Table 1). Future challenges in experimental design include the development of sequencing and analysis tools that are better designed to identify complex genomic rearrangements and gene duplication, as one recent paper elegantly demonstrated that copy number expansion can be a powerful driving force in the rapid adaptation of viruses to a suboptimal host [25]. Furthermore, as real-world pathogen adaptation is never as simple as one host, one pathogen, a logical next step will be to include coinfections with multiple pathogens [26] in these laboratory models to allow for interspecies competition as well as horizontal gene transfer between adapting pathogens. Lastly, as the price of sequencing continues to fall, studying the host genome's response to prolonged pathogen exposure will be a natural extension for the field.

**Table 1:** Considerations in designing a laboratory passage experiment.

Consideration	Why it matters
Population size	Too small and genetic drift can result in mutations that reduce overall fitness.
Mutation rate	Too high (through the use of mutagens) and the number of background hitchhiker mutations make linking phenotype to genotype more difficult.
Choosing isolates to sequence	The existence of complex population dynamics, such as clonal interference, means that individual clones from individual time-points represent only a snapshot. Sequencing intermediates and measuring allelic frequencies in the evolving population may give a more complete picture of evolutionary trajectories.
When to stop	Fitness of a population, in general, increases at a greater rate per generation early during passage, though open-ended experiments continue to lead to surprising results decades after they began.

## References

[1] Lenski RE, Travisano M (1994) Dynamics of adaptation and diversification: a 10,000-generation experiment with bacterial populations. Proc Natl Acad Sci U S A 91: 6808–6814. 8041701 10.1073/pnas.91.15.6808 PMC44287

[2] Hyman ED (1988) A new method of sequencing DNA. Anal Biochem 174: 423–436. 2853582 10.1016/0003-2697(88)90041-3

[3] Wetterstrand K. DNA sequencing costs: data from the NHGRI Genome Sequencing Program (GSP). Available: http://www.genome.gov/sequencingcosts/. Accessed 29 April 2013.

[4] Kawecki TJ, Lenski RE, Ebert D, Hollis B, Olivieri I, et al (2012) Experimental evolution. Trends Ecol Evol 27: 547–560. 22819306 10.1016/j.tree.2012.06.001

[5] Sabin AB, Schlesinger RW (1945) Production of immunity to dengue with virus modified by propagation in mice. Science 101: 640–642. 17844088 10.1126/science.101.2634.640

[6] Ebert D (1998) Experimental evolution of parasites. Science 282: 1432–1435. 9822369 10.1126/science.282.5393.1432

[7] Kassen R (2002) The experimental evolution of specialists, generalists, and the maintenance of diversity. J Evol Biol 15: 173–190.

[8] Cooper VS, Lenski RE (2000) The population genetics of ecological specialization in evolving Escherichia coli populations. Nature 407: 736–739. 11048718 10.1038/35037572

[9] Ensminger AW, Yassin Y, Miron A, Isberg RR (2012) Experimental evolution of Legionella pneumophila in mouse macrophages leads to strains with altered determinants of environmental survival. PLoS Pathog 8: e1002731 doi:10.1371/journal.ppat.1002731 22693450 10.1371/journal.ppat.1002731 PMC3364954

[10] Fields BS (1996) The molecular ecology of legionellae. Trends Microbiol 4: 286–290. 8829338 10.1016/0966-842x(96)10041-x

[11] Ciota AT, Kramer LD (2010) Insights into arbovirus evolution and adaptation from experimental studies. Viruses 2: 2594–2617. 21994633 10.3390/v2122594 PMC3185588

[12] Deardorff ER, Fitzpatrick KA, Jerzak GVS, Shi P-Y, Kramer LD, et al (2011) West Nile virus experimental evolution in vivo and the trade-off hypothesis. PLoS Pathog 7: e1002335 doi:10.1371/journal.ppat.1002335 22102808 10.1371/journal.ppat.1002335 PMC3213084

[13] Herfst S, Schrauwen EJ, Linster M, Chutinimitkul S, de Wit E, et al (2012) Airborne transmission of influenza A/H5N1 virus between ferrets. Science 336: 1534–1541. 22723413 10.1126/science.1213362 PMC4810786

[14] Imai M, Watanabe T, Hatta M, Das SC, Ozawa M, et al (2012) Experimental adaptation of an influenza H5 HA confers respiratory droplet transmission to a reassortant H5 HA/H1N1 virus in ferrets. Nature 486: 420–428. 22722205 10.1038/nature10831 PMC3388103

[15] Ebert D, Bull JJ (2003) Challenging the trade-off model for the evolution of virulence: is virulence management feasible? Trends Microbiol 11: 15–20. 12526850 10.1016/s0966-842x(02)00003-3

[16] Pandya U, Sinha M, Luxon BA, Watson DA, Niesel DW (2009) Global transcription profiling and virulence potential of Streptococcus pneumoniae after serial passage. Gene 443: 22–31. 19397959 10.1016/j.gene.2009.04.014

[17] Traverse CC, Mayo-Smith LM, Poltak SR, Cooper VS (2013) Tangled bank of experimentally evolved Burkholderia biofilms reflects selection during chronic infections. Proc Natl Acad Sci U S A 110: E250–259. 23271804 10.1073/pnas.1207025110 PMC3549113

[18] Almahmoud I, Kay E, Schneider D, Maurin M (2009) Mutational paths towards increased fluoroquinolone resistance in Legionella pneumophila. J Antimicrob Chemother 64: 284–293. 19474069 10.1093/jac/dkp173

[19] Anderson JB, Sirjusingh C, Parsons AB, Boone C, Wickens C, et al (2003) Mode of selection and experimental evolution of antifungal drug resistance in Saccharomyces cerevisiae. Genetics 163: 1287–1298. 12702675 10.1093/genetics/163.4.1287 PMC1462505

[20] Wong A, Rodrigue N, Kassen R (2012) Genomics of adaptation during experimental evolution of the opportunistic pathogen Pseudomonas aeruginosa. PLoS Genet 8: e1002928 doi:10.1371/journal.pgen.1002928 23028345 10.1371/journal.pgen.1002928 PMC3441735

[21] Lieberman TD, Michel JB, Aingaran M, Potter-Bynoe G, Roux D, et al (2011) Parallel bacterial evolution within multiple patients identifies candidate pathogenicity genes. Nat Genet 43: 1275–1280. 22081229 10.1038/ng.997 PMC3245322

[22] Yang L, Jelsbak L, Marvig RL, Damkiaer S, Workman CT, et al (2011) Evolutionary dynamics of bacteria in a human host environment. Proc Natl Acad Sci U S A 108: 7481–7486. 21518885 10.1073/pnas.1018249108 PMC3088582

[23] Zdziarski J, Brzuszkiewicz E, Wullt B, Liesegang H, Biran D, et al (2010) Host imprints on bacterial genomes—rapid, divergent evolution in individual patients. PLoS Pathog 6: e1001078 doi:10.1371/journal.ppat.1001078 20865122 10.1371/journal.ppat.1001078 PMC2928814

[24] Herron MD, Doebeli M (2013) Parallel evolutionary dynamics of adaptive diversification in Escherichia coli. PLoS Biol 11: e1001490 doi:10.1371/journal.pbio.1001490 23431270 10.1371/journal.pbio.1001490 PMC3576414

[25] Elde NC, Child SJ, Eickbush MT, Kitzman JO, Rogers KS, et al (2012) Poxviruses deploy genomic accordions to adapt rapidly against host antiviral defenses. Cell 150: 831–841. 22901812 10.1016/j.cell.2012.05.049 PMC3499626

[26] Leggett HC, Benmayor R, Hodgson DJ, Buckling A (2013) Experimental evolution of adaptive phenotypic plasticity in a parasite. Curr Biol 23: 139–142. 23246405 10.1016/j.cub.2012.11.045

